# Microvascular blood flow ultrasound imaging with microbubble-based H-Scan technology

**DOI:** 10.1007/s11517-024-03262-1

**Published:** 2025-01-21

**Authors:** Feng Jiang, Yiheng Li, Yaoyao Cui, Yang Jiao

**Affiliations:** 1 School of Biomedical Engineering (Suzhou), Division of Life Sciences and Medicine, University of Science and Technology of China, Suzhou, 230026 China; 2 Suzhou Institute of Biomedical Engineering and Technology, Chinese Academy of Science, Suzhou, 215613 China

**Keywords:** Microbubbles, H-Scan, Microvascular ultrasound imaging, Gaussian weighted Hermite polynomials, Signal-to-noise ratio

## Abstract

Ultrasound blood flow imaging plays a crucial role in the diagnosis of cardiovascular and cerebrovascular diseases. Conventional ultrafast ultrasound plane-wave imaging techniques have limited capabilities in microvascular imaging. To enhance the quality of blood flow imaging, this study proposes a microbubble-based H-Scan ultrasound imaging technique. This technique utilizes high-order H-Scan to detect the Rayleigh scattering contributed by blood flow and microbubbles at certain concentrations. The detected results are then processed in the B channel using methods such as clutter filtering based on Casorati matrix singular value decomposition (Casorati-SVD). Compared with the control group without H-Scan, the signal-to-noise ratio (SNR) of the experimental group using the microbubble-based H-Scan ultrasound imaging technique was increased by 38.61% on average and the contrast signal-to-noise ratio (CNR) was increased by 39.5% on average. The improved image quality of microvascular flow imaging was visibly enhanced. This method demonstrates significant advantages in enhancing the sensitivity and accuracy of ultrasound blood flow imaging, indicating considerable potential for clinical applications.

**Graphical abstract:**

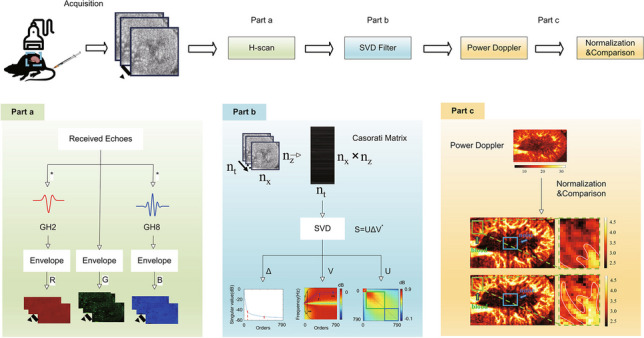

## Introduction

Ultrasound blood flow imaging is a crucial diagnostic technique in the medical field. Color Doppler imaging (CDI) utilizes frequency analysis to display the velocity and direction of blood flow [32]. Pulsed Doppler imaging utilizes narrow pulse sound waves to locate and detect blood flow velocity at specific vessels [21]. Power Doppler imaging is a high-sensitivity blood flow imaging technique capable of detecting slower blood flow velocities and displaying the distribution of blood flow [28]. Ultrafast ultrasound systems utilize plane wave ultrasound with high frame rates to greatly advance ultrasound blood flow imaging, particularly with ultrafast Doppler ultrasound enabling the imaging of microvascular flow [4, 27, 35]. With the development of microbubbles, contrast-enhanced ultrasound (CEUS) has become increasingly important in blood flow imaging, significantly enhancing the ultrasound signal and improving both the contrast and clarity of the image [1]. The method is widely used in the diagnosis of diseases [13, 20, 36]. Over the past decade, ultrasound localization microscopy (ULM) has been extensively researched. It achieves super-resolution imaging by detecting, localizing, and tracking injected microbubbles, offering enhanced sensitivity and safety [9]. The method has been successful in imaging the kidney, brain, spinal cord, and other parts of the body. At typical microbubble concentrations, the microbubble cloud can be considered a subwavelength random distribution of Rayleigh scattering [8, 11, 12, 15, 18].

Clutter filtering is essential in ultrasound blood flow imaging to distinguish Doppler signals from moving red blood cells from low-frequency signals generated by stationary tissue reflections, known as clutter, and noise. Traditional clutter suppression typically employs finite impulse response (FIR) or infinite impulse response (IIR) filters with fixed cutoff frequencies for high-pass filtering. Both types filter based on the principle that clutter signals, which are typically 40–80 dB stronger than blood flow signals, are attenuated. This method predominantly utilizes the temporal characteristics of ultrasound echo signals, neglecting the spatial coherence of ultrasound data, which may lead to loss of information regarding slow blood flow [5]. Demené et al. proposed a clutter filtering algorithm based on Casorati matrix singular value decomposition (Casorati-SVD) in 2015, leveraging distinct spatiotemporal statistical properties of tissue and blood signals [10]. The Casorati-SVD clutter filtering utilizes two threshold values to separate tissue, blood, and noise subspaces. Therefore, precise determination of these thresholds is crucial for the Casorati-SVD clutter filtering. Current methods for determining the two thresholds include the following:

1. Setting two thresholds based on experience. This method lacks robustness.
2. Singular value-based estimation: Determining the choice of the low-order threshold is done when the ratio of the cumulative sum of the blood singular values to the total signal energy (defined as the sum of all singular values) reaches a certain (negative) dB level. And the higher order threshold can be determined according to the Marchenko-Pastur distribution.
3. Time singular vector-based estimation: Selecting thresholds based on the PSD-weighted central frequency, standard deviation, and bandwidth.
4. Spatial singular vector-based estimation: Defining cutoff thresholds by the boundaries of three highly correlated matrices appearing in the non-coherent correlation matrix of spatial singular vectors.

The Casorati-SVD clutter filtering enhances filtering efficacy compared to traditional filtering, enabling the detection of more micro-vessels and slow blood flow [3, 30, 31].

H-Scan is a technique capable of estimating the relative size and spatial distribution of acoustic scatterers [29]. The technique can classify the echo signal into large scatterers, thin scatterers, and Rayleigh scatterers. The method has shown promising results in detecting thyroid lesions and identifying early tumors [14, 16, 23–25, 33, 34]. The higher-order H-Scan technique has been shown to have potential for the detection of red blood cells.

This paper proposes combining the H-Scan method with microbubble imaging to utilize the ability of the H-Scan to distinguish tissue scattering features within the field of view to further improve the quality of blood flow imaging. The H-Scan processed envelope detection results are RGB color-coded. Specifically, ultrasound signals containing large-scale scatterers are input into the R channel (signal information mapped as red), while the original signals without H-Scan processing are input into the G channel (signal information mapped as green). The Rayleigh scattering signals contributed by blood flow and microbubbles of specific concentrations are input into the B channel (signal information mapped as blue). The B channel is extracted for subsequent processing, such as Casorati-SVD clutter filtering, to obtain high-quality blood flow imaging results.

## Theory and method

### H-Scan ultrasound imaging

H-Scan ultrasound imaging is a novel imaging $q$technique capable of estimating the relative size and spatial distribution of acoustic scatterers. The technique closely relates Gaussian-weighted ergodic functions to large layer scatterers, thin layer scatterers, and Rayleigh scatterers (such as red blood cells) [29].

Ultrasound echo signals *e*(*t*) can be represented by a convolutional model:

1 $$e\left(t\right)=A\left\{p\left(t\right)s\left(x,y\right)***R\left(x,y,\frac{ct}{2}\right)\right\}$$

where *A* is the amplitude constant, *p*(*t*) is the ultrasound pulse propagating along the axial direction, *s*(*x*, *y*) is the beam profile (beam width along the lateral and elevation axes), *R* represents the three-dimensional pattern of reflectors or scatterers, *c* is the speed of sound, and *** denotes the three-dimensional convolution.

Assuming the spatial variation of acoustic impedance *Z* = *ρc* is small, the function *R* can be correlated with the spatial derivative of acoustic impedance along the imaging pulse propagation direction *z*, expressed as follows:

2 $$R\left(z\right)\approx \left(\frac{1}{2Z}\right)\frac{dZ\left(z\right)}{dz}$$

The above equation shows that specific structures produce characteristic reflections. For thin layers with thin interfaces and high acoustic impedance, a positive pulse is generated on the front surface and a negative pulse on the back surface. In the limiting case, it approaches a Dirac delta-like function, represented by its derivative *δ'*(*t*). In this scenario, the returned echo is proportional to the derivative of *p*(*t*) with respect to time, indicating frequency content weighted by *ω*. For large layers, the returned echo is proportional to *p*(*t*) and can be represented by *δ*(*t*), indicating frequency content weighted by 1. When the acoustic scattering source is smaller than the ultrasonic wavelength, Rayleigh scattering occurs. For small (sub-wavelength) spherical scatterers, the dominant term in the scattered pressure under the Born approximation is proportional to *ω*^2^. Additionally, a group of small, weak scatterers arranged in a disordered cloud-like structure also exhibits a scattered pressure dependence with a dominant term proportional to *ω*^2^. According to the Fourier transform theorem, the *ω*^2^ weighting corresponds to the second derivative of the function [7, 19].

3 $$\mathcal{F}\left\{\frac{{d}^{2}p\left(t\right)}{d{t}^{2}}\right\}\to {\omega }^{2}P\left(\omega \right)$$

Here, *F{}* denotes the Fourier transform operation, and *P*(*ω*) represents the Fourier transform of *p*(*t*). Consequently, it can be inferred that the echo from Rayleigh scattering is proportional to *d*^2^*p*/*dt*^2^.

Parker relates Gaussian-weighted Hermite polynomials to the three classes of ultrasonic scatterers mentioned above. The Hermite polynomials are defined as follows:

4 $${H}_{n}\left(t\right)={\left(-1\right)}^{n}{e}^{{t}^{2}}\frac{{d}^{n}}{d{t}^{n}}{e}^{{-t}^{2}}$$

where *n* = 0, 1, 2,..., *t* ∈ (− ∞, + ∞), when Eq. 4 is multiplied by the continuous derivative of the Gaussian pulse *G*(*t*) = *e*^*−t*2^, the Gaussian-weighted Hermite polynomials are obtained. Table 1 presents the first nine orders of Gaussian-weighted Hermite polynomials (GHs). The relationship between GHs and the time derivatives is given by the following:

5 $$\frac{d}{dt}\left(G\bullet {H}_{n}\left(t\right)\right)=-G\bullet {H}_{n+1}\left(t\right)$$

where *n* = 0, 1, 2,...,* t* ∈ (− ∞, + ∞).

**Table 1:** Gaussian-weighted Hermite polynomials

Order, *n*	GHn(*t*)	*E*(*n*)
0	$\left(1\right){e}^{-t}$	√*(π*/2*)*
1	$\left(2t\right){e}^{-t}$	√*(π*/2*)*
2	$\left(4{t}^{2}-2\right){e}^{{-t}^{2}}$	3√*(π*/2*)*
3	$\left(8{t}^{3}-12t\right){e}^{{-t}^{2}}$	15√*(π*/2*)*
4	$\left(16{t}^{4}-48{t}^{2}+12\right){e}^{{-t}^{2}}$	105√*(π*/2*)*
5	$\left(32{t}^{5}-160{t}^{3}+120t\right){e}^{{-t}^{2}}$	945√*(π*/2*)*
6	$\left(64{t}^{6}-480{t}^{4}+720{t}^{2}-120\right){e}^{{-t}^{2}}$	10,395√*(π*/2*)*
7	$\left(128{t}^{7}-1344{t}^{5}+3360{t}^{3}-1680t\right){e}^{{-t}^{2}}$	135,135√*(π*/2*)*
8	$\left(256{t}^{8}-3584{t}^{6}+13440{t}^{4}-13440{t}^{2}+1680\right){e}^{{-t}^{2}}$	2,027,025√*(π*/2*)*

*E*(*n*) represents the energy of GHn(*t*), and the equation is expressed as follows: $E\left(n\right)={\int }_{-\infty }^{\infty }{G}^{2}{H}_{n}^{2}dt=1\times 3\times 5\times \dots \times \left|2n-1\right|\times \sqrt{\pi /2},n=\text{0,1,2,}\dots ,t\in \left(-\infty ,+\infty \right)$

**Fig. 1:**
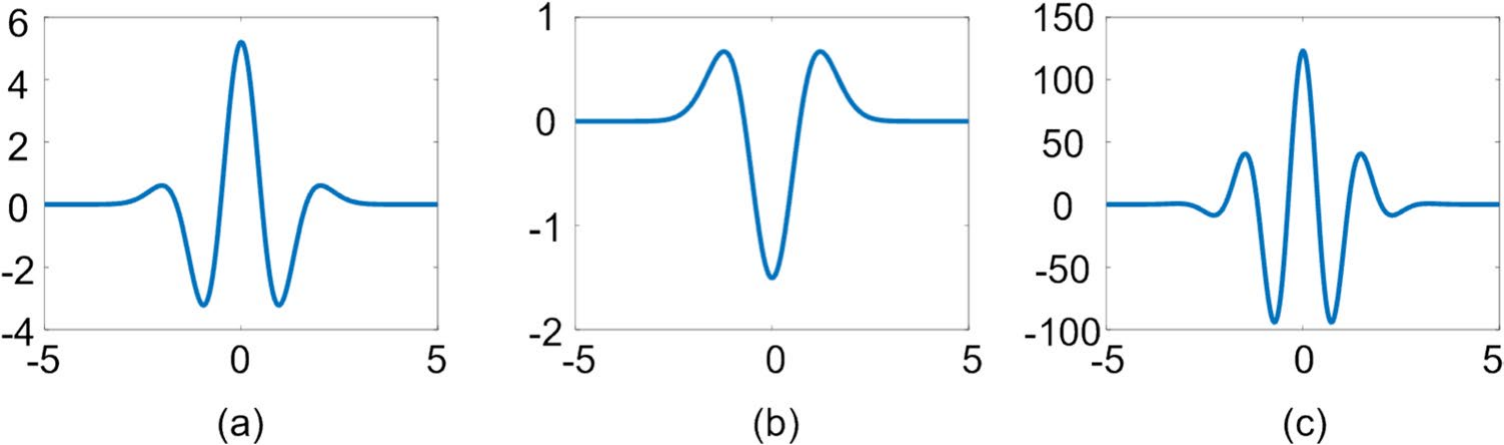
**a** GH_4_ kernel. **b** GH_2_ kernel. **c** GH_8_ kernel

A typical broadband ultrasound transmission signal can be approximated as a GH_4_ function (the kernel is shown in Fig. 1a). Assuming that the round-trip impulse response of the pulse-echo system is *p*(*t*) = *A*_0_*GH*_4_(*t*)$p\left(t\right)={A}_{0}G{H}_{4}(t)$, then the signals received by large layer reflection, thin layer reflection, and Rayleigh scattering can be written as respectively:

6 $$e_{\mathrm{Large}}\left(t\right)=\left(A_0GH_4\left(t\right)\right)\ast R_0\delta\left(t\right)=R_0A_0GH_4\left(t\right)$$

7 $$e_{\mathrm{Thin}}\left(t\right)=\left(A_0GH_4\left(t\right)\right)\ast R_1\delta\left(t\right)=R_1A_0GH_5\left(t\right)$$

8 $$e_{\mathrm{Rayleigh}}\left(t\right)=\left(A_0GH_4\left(t\right)\right)\ast R_2\delta\left(t\right)=R_2A_0GH_6\left(t\right)$$

In the framework of the aforementioned formulas, the identification task involves simply categorizing based on the similarity between echoes and *GH*_4_(*t*), *GH*_5_(*t*), or *GH*_6_(*t*). To achieve effective classification, matched filters such as parallel or maximum likelihood filters can be utilized. This involves convolving the received signal with scaled versions of *GH*_4_(*t*), *GH*_5_(*t*), and *GH*_6_(*t*) to generate three post-processed signals. Two parallel convolution filter outputs (*GH*_4_(*t*) and *GH*_6_(*t*)) are allocated to the R and B channels, respectively, to measure the relative intensity of large layers and Rayleigh scatter reflections. In practice, emphasizing spectral peaks more, such as normalizing by *√E*_*n*_ to reduce the correlation between overlapping GHs spectra like *GH*_2_(*t*) and *GH*_8_(*t*) (the kernel is shown in Fig. 1b and c), the parallel convolution results are inputted into the R and B channels [22, 29].

### Microbubble-based H-Scan ultrasound imaging

Microbubbles are tiny gas bubbles made of biocompatible substances that are typically between 1 and 10 µm in diameter. The second-generation commercial microbubble contrast agent, SonoVue, approved for diagnostic applications in the USA, Europe, and China, consists of sulfur hexafluoride gas encapsulated within a phospholipid monolayer shell [6, 17, 26]. Due to the strong compressibility of the sulfur hexafluoride gas inside the microbubbles, they have much higher acoustic impedance than red blood cells (which are weakly compressible), resulting in stronger ultrasound echo signals [2]. The average diameter of red blood cells is 7.2 µm [22]. The size of microbubbles is similar to that of red blood cells, and since the source of acoustic scattering for both is smaller than the ultrasonic wavelength, Rayleigh scattering occurs. In the H-Scan ultrasound imaging technique, the convolution result of the input B channel with a higher order Gaussian-weighted Hermit polynomial is better able to detect the relative intensity of the reflections from the Rayleigh scatterers. After the injection of microbubbles, the B channel of the H-Scan ultrasound imaging technique demonstrated the potential to improve microvascular resolution.

**Fig. 2:**
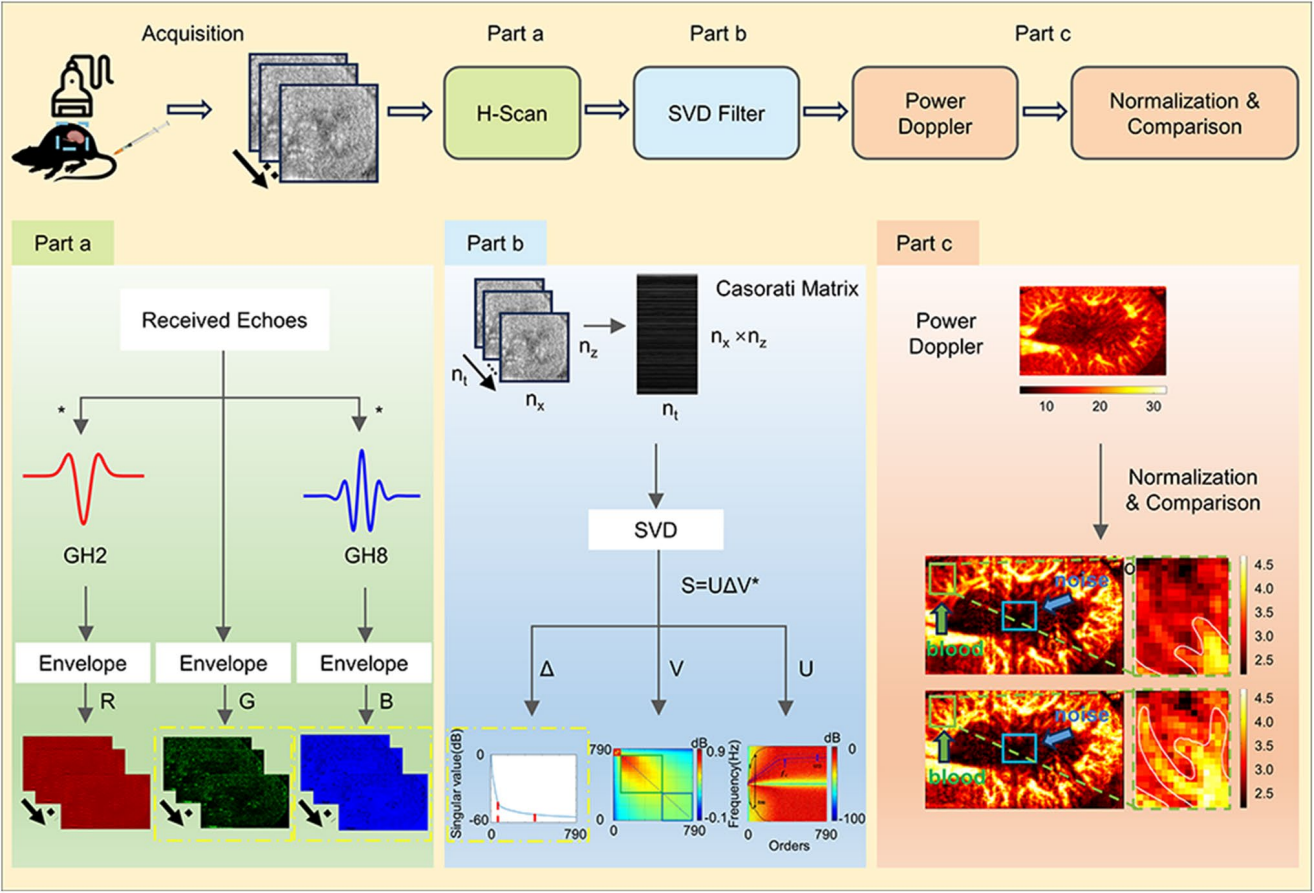
Flowchart. The upper part of the figure shows the overall flowchart, and the lower part shows the detailed steps of some of the processes. Part **a** shows the flowchart of microbubble-based H-Scan ultrasound imaging processing, part **b** shows the flowchart of Casorati-SVD filtering processing, and part **c** shows the power Doppler imaging and the normalization and comparison part

Accordingly, in this paper, a microbubble-based H-Scan ultrasound imaging technique is proposed by combining microbubbles with H-Scan ultrasound imaging, aiming to further enhance the ultrasound imaging capability of micro-vessels. The flow of the technique is shown in Fig. 2. Firstly, the ultrasound RF signals of the sample were acquired and subjected to beam synthesis, and the beamformed data were subsequently subjected to microbubble-based H-Scan ultrasound imaging processing, whereby the lower-order convolved results were input into the R channel, the un-convolved results into the G channel, and the higher-order convolved results were input into the B channel (see Fig. 2, part a, for details). Next, the G channel and B channel data responding to thin layer scattering and Rayleigh scattering, respectively, were used for Casorati-SVD filtering processing, attempting to estimate the high and low cutoff thresholds from the two directions of singularity values and spatial singular vectors, respectively, and selecting the filtering result with the best effect among them for the subsequent processing (see Fig. 2, part b, for details). After filtering, power Doppler imaging was performed, and in order to compare the power Doppler imaging results, we normalized the power Doppler display range and selected the region of interest (ROI) of the blood flow and the background noise ROI on the normalized images in order to compare the power Doppler images before and after the use of the microbubble-based H-Scan ultrasound imaging technique. Finally, according to Eqs. 9 and 10, the signal-to-noise ratio (SNR) and contrast-signal-to-noise ratio (CNR) were calculated to quantitatively evaluate the effect of the imaging algorithm (see Fig. 2, part c, for details):

9 $$SNR=\frac{{\overline{s}}_{blood}}{{\sigma }_{noise}}$$

10 $$CNR=\frac{{\overline{s}}_{blood}-{\overline{s}}_{tissue}}{{\sigma }_{noise}}$$

where‾*s*_blood_ is the average blood flow signal, *σ*_noise_ is the noise standard deviation, and‾*s*_tissue_ is the mean value of background tissue signal around blood vessels. A high signal-to-noise ratio means that the intensity of the blood flow signal in the image is stronger than the noise signal, and a high signal-to-noise ratio means that the blood flow signal in the image is stronger than the background noise, which generally reflects a higher image quality.

## Experimental setup

### Phantom experiment

**Fig. 3:**
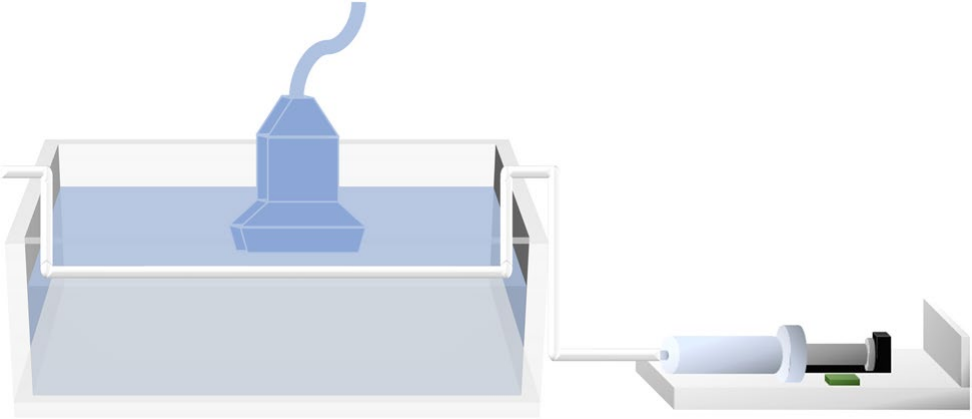
Diagram of the body-mimicking experimental setup

A plastic tube with an outer diameter of approximately 1.86 mm was horizontally placed in water to simulate a blood vessel. Microbubbles were prepared using SonoVue contrast agent (Bracco Research, Switzerland), where lyophilized powder was mixed with 5 mL of saline solution (0.9% NaCl) to form a suspension with a microbubble concentration of 8 µL/mL (average diameter of 2.5 µm). The suspension was manually infused into the tube at a rate of 300 µL every 30 s. A laptop-based ultrasound scanner equipped with a linear array transducer (L25-15L20N-3) operating at a central frequency of 10 MHz was used to detect microbubbles parallel to the tube; the experimental setup diagram is shown in Fig. 3. H-Scan processing was applied separately to two sets of phantoms—one with injected microbubbles and the other without—to compare the experimental outcomes.

### Animal experiment

This study validated the microbubble-based H-Scan technique using publicly available rat kidney data from the PALA dataset. Rats were immobilized, and microbubbles (SonoVue contrast agent, Bracco Imaging, Milan, Italy) were manually infused via the tail vein at a rate of 50 µL every 30 s. Ultrasound IQ data acquisition utilized a linear transducer (L22-14v, central frequency 15.625 MHz, Vantage 256, Verasonics, USA), employing coherent compounding with five plane waves (angles, − 10°, − 5°, 0°, 5°, and 10°) at a frame rate of 1 kHz. In this study, eight randomly selected datasets were processed for subsequent analysis, comparing experimental outcomes with and without H-Scan processing.

## Result

### Phantom experiment

**Fig. 4:**
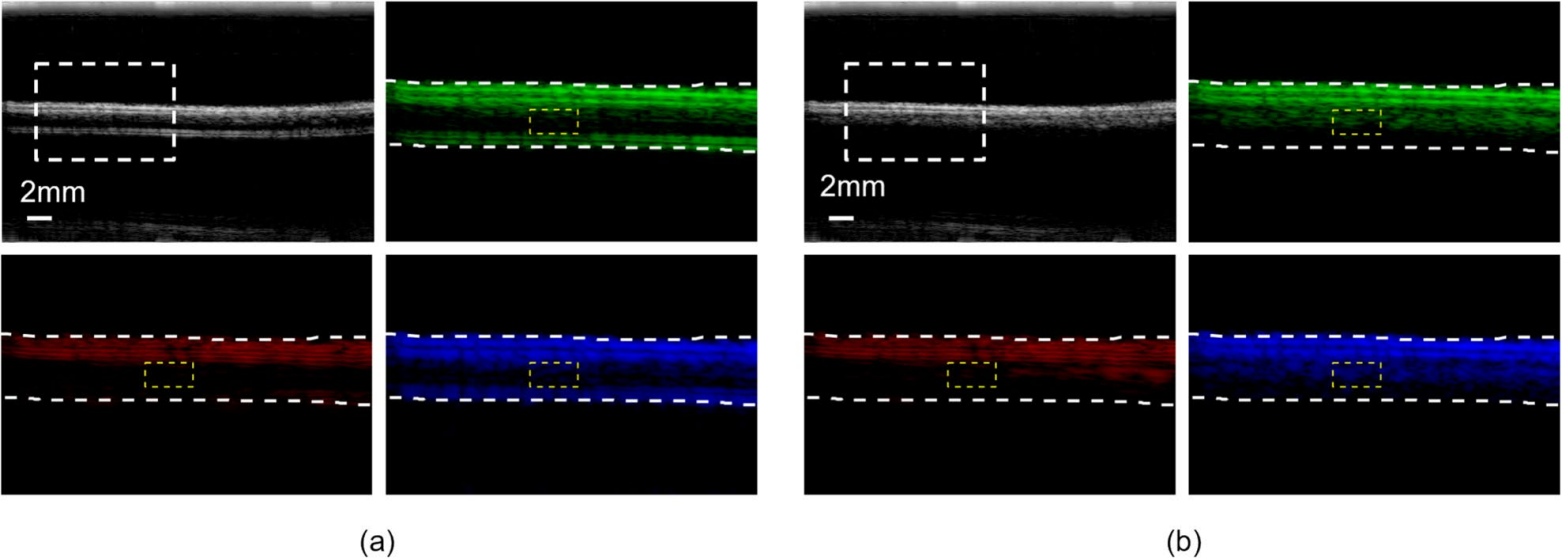
**a** Non-injected microbubble group: B-mode ultrasound images locally magnified around the white dashed lines after H-Scan processing, with results input into R, G, and B channels. Yellow dashed lines indicate the selected ROI within the tube. **b** Microbubble-injected group: B-mode ultrasound images locally magnified around the white dashed lines after H-Scan processing, with results input into R, G, and B channels. Yellow dashed lines indicate the selected ROI within the tube

We validated the effectiveness of the microbubble-based H-Scan ultrasound imaging technique through phantom experiments. Upon microbubble injection into the tube, there was a notable increase in signal intensity within the tube, facilitating clear visualization of its contents. Both microbubble-injected and non-injected phantom groups underwent H-Scan processing. The original B-mode images were locally magnified around the white dashed lines. Low-order convolution results (envelope detection results convolved with GH_2_) were input into the R channel, revealing higher signal intensity uniformly along the phantom walls, with lesser intensity on the intratubal microbubbles. High-order convolution results (envelope detection results convolved with GH_8_) were input into the B channel, showing higher signal intensity specifically on the intratubal microbubbles. Results without convolution (envelope detection of the original signal) were input into the G channel. A 1 mm × 2 mm ROI (indicated by yellow dashed lines) was selected within the tube to evaluate changes in signal intensity before and after employing the H-Scan technique. In Fig. 4a, the ROI intensity in the R channel for non-injected microbubbles decreased, possibly due to artifacts during acquisition, with no significant change observed in the B channel ROI. In Fig. 4b, the ROI intensity in the R channel for injected microbubbles decreased by 96.93%, while the ROI intensity in the B channel increased by 202.66%.

Selecting an appropriate order of Gaussian-weighted Hermite polynomials in the H-Scan technique can effectively enhance the signal intensity of microbubbles (Rayleigh scattering) at a certain concentration.

### Animal experiment

To assess the efficacy of microbubble-based H-Scan ultrasound imaging for in vivo imaging, we used an open dataset of rat kidneys injected with microbubbles and randomly selected eight sets of experimental data to compare power Doppler results with and without H-Scan ultrasound imaging.

**Fig. 5:**
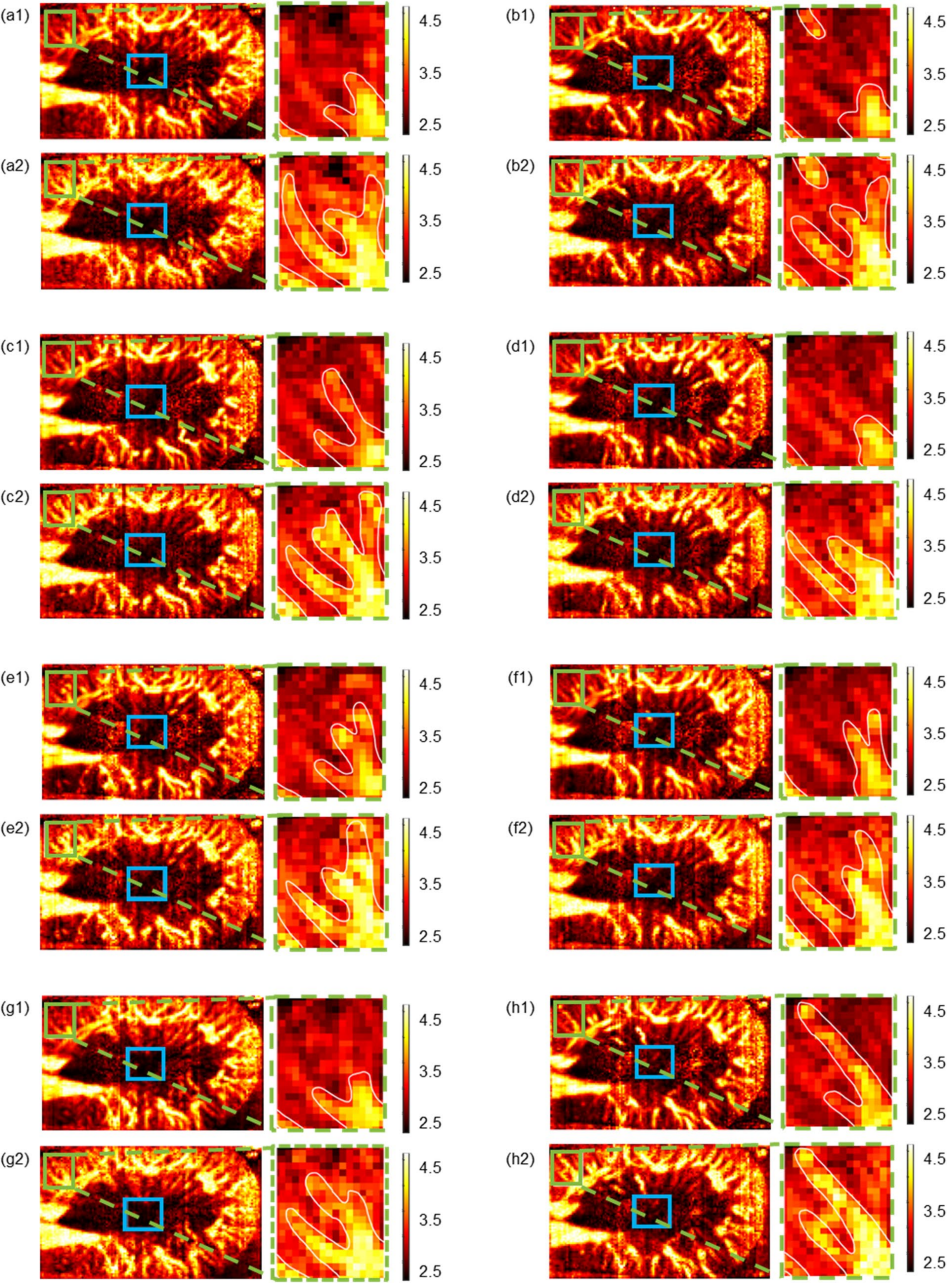
Power Doppler images of rat kidney. Eight sets of experimental data with power Doppler imaging were categorized into eight groups: a, b, c, d, e, f, g, and h. In each set of figures, label 1 at the top represents the original microbubble-only power Doppler image (the power Doppler image processed from G channel data), and label 2 at the bottom represents the power Doppler image with microbubble injection and H-scan processing (the power Doppler image processed from B channel data). The blue box represents selected background noise ROI; the green box represents selected section containing small blood vessels, and the magnified image is on the right, with the location of the blood flow circled in white

The experimental results are shown in Fig. 5, where the image labeled 1 shows the power Doppler image without using the microbubble-based H-Scan ultrasound imaging technique and the image labeled 2 shows the power Doppler image using the microbubble-based H-Scan ultrasound imaging technique. In the image, the blue box line marks the selected background noise ROI, while the green box line marks the selected blood flow ROI. By zooming in on the selected blood flow ROI, we can observe that the imaging quality of the tiny blood flow can be significantly improved using the microbubble-based H-Scan ultrasound imaging technique. Based on the signal strength of the ROI, SNR and CNR were calculated and the results are shown in Table 2. The results showed that SNR was improved by 10.35–73.84% and CNR was improved by 15.46–77.49%. The microbubble-based H-Scan ultrasound imaging method can effectively improve the quality of blood flow imaging.

**Table 2:** Comparison of SNR and CNR of rat kidney Doppler ultrasound images before and after using microbubble-based H-Scan ultrasound imaging techniques

Group	Before			After			Increase ratio of SNR	Increase ratio of CNR
	SVD threshold	SNR (dB)	CNR (dB)	SVD threshold	SNR (dB)	CNR (dB)		
a	[31,782]	75.55	53.78	[21,785]	88.85	62.45	17.60%	16.12%
b	[39,779]	29.53	41.43	[26,782]	46.42	67.69	63.36%	57.22%
c	[38,784]	60.67	43.77	[27,780]	85.14	61.68	63.36%	57.22%
d	[36,782]	39.68	25.93	[31,786]	68.99	46.02	73.84%	77.49%
e	[42,784]	65.03	43.47	[24,782]	71.76	50.19	10.35%	15.46%
f	[31,786]	43.10	28.80	[21,789]	50.37	33.65	16.87%	16.83%
g	[32,785]	55.75	35.13	[22,782]	71.59	49.27	28.41%	40.26%
h	[31,786]	47.57	31.31	[21,789]	64.26	42.39	35.08%	35.40%

*Before* refers to the results obtained prior to using microbubble-based H-Scan ultrasound imaging techniques.

*After* refers to the results obtained after the use of the microbubble-based H-Scan ultrasound imaging technique.

*SVD threshold* refers to the two thresholds for the selected suitable Casorati-SVD filtering (estimated based on singular values).

## Discussion

This paper proposes combining H-Scan technology, capable of estimating relative size and spatial distribution of acoustic scatterers, with microbubble injection to improve the imaging quality of the microvascular system. Phantom experiments demonstrate the superiority of H-Scan technology in detecting microbubbles at specific concentrations. Animal experiments utilized B channel data from GH8 convolution outputs, which are more sensitive to Doppler shifts from blood flow and certain microbubble concentrations, processed with Casorati-SVD clutter filtering. Subsequently, microbubble tracking and power Doppler imaging were conducted. Quantitative analysis of signal-to-noise ratio and local signal-to-noise ratio revealed that this method effectively improves imaging quality of microvascular flow.

### Improvement of CNR in phantom experiments

**Fig. 6:**
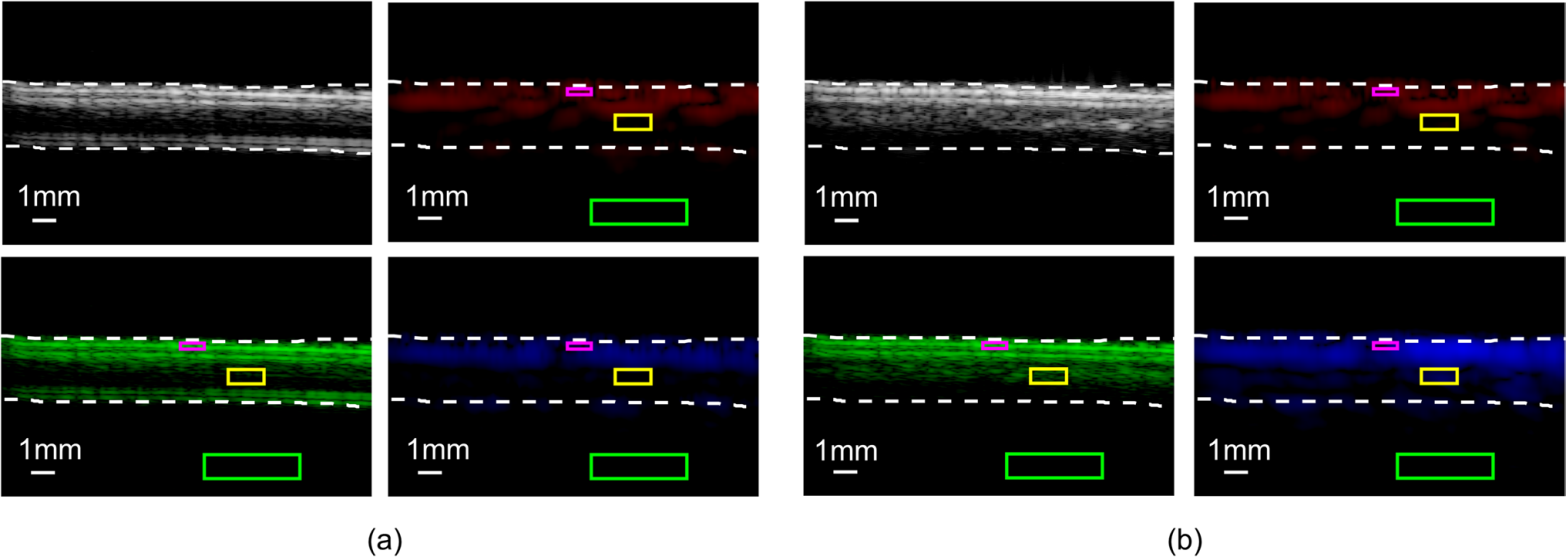
Phantom experiments for CNR comparison. The yellow box is the blood flow signal ROI, the red box is the tissue signal ROI, and the green box is the background noise signal ROI. **a** Non-injected microbubble group. **b** Microbubble-injected group. Original results are input into the G channel, low-order H-Scan results are input to the R channel, and high-order H-Scan results are input to the B channel

In phantom experiments, we verified that the higher-order H-Scan technique was specific for microbubbles from another direction using CNR. We performed H-Scan on the non-injected microbubble group and the injected microbubble group separately. The convolution results with higher-order GHs were input into the B channel, the convolution results with lower-order GHs were input into the R channel, and the unconvolved results were input into the G channel. As shown in Fig. 6, the yellow boxed area was used to calculate‾*s*_blood_, the red boxed area was used to calculate*‾s*_tissue_, and the green boxed area was used to calculate *σ*_noise_. The results of CNR calculation are shown in Table 3. It was found that the group without injecting bubbles had no significant change in‾*s*_blood_ after H-Scan treatment. For the group injected with bubbles, the CNR was significantly increased to 46.56 dB after the higher-order H-Scan treatment.*‾s*_blood_ was increased to some extent and‾*s*_tissue_ was decreased to some extent in the B channel results of the group compared to the G channel results. The results suggest that higher-order H-Scan can detect a certain concentration of microbubbles. In other words, the microbubble-based H-Scan technique utilized in this paper is more advantageous in CNR compared to the H-Scan-only technique.

**Table 3:** Phantom experiments for CNR comparison

	Non-injected microbubble			Microbubble-injected		
	R channel	G channel	B channel	R channel	G channel	B channel
‾*s*_blood_	9.46	10.57	9.88	13.78	32.83	40.78
‾*s*_tissue_	43.48	35.66	19.79	41.88	39.78	17.50
*σ*_noise_	0.54	0.49	0.47	0.55	0.56	0.50
CNR (dB)	− 63.00	− 51.21	− 21.07	− 51.09	− 13.90	46.56

### Evaluation of Casorati-SVD filter thresholds

In this paper, when selecting the threshold value of the Casorati-SVD filter, we consider estimating it from two directions: the spatial singular value vector and the singular value. When estimating the threshold value based on the spatial singular value vector, in a near-ideal model environment, the tissue and blood flow of the rat kidney hardly overlap, and the correlation matrix of the spatial singular value can clearly show two diagonally adjacent correlation squares of the tissue and blood flow. However, in this experiment, the different spatial subspaces of the correlation matrix of spatial singular values are not clearly displayed, which leads to the difficulty and computationally intensive to go to find the boundary of the squares. Therefore, we choose to estimate the thresholds of the Casorati-SVD filter from the singular value direction, with the lower order thresholds determined by the horizontal coordinates corresponding to the first local minima of the radius of curvature of the singular value curve and the higher order thresholds determined based on the Gaussian noise signal following the Marchenko-Pastur distribution. The thresholds selected in the animal experiments are given by the “SVD threshold” in Table 2.

In Table 2, it can be seen that the low-order thresholds of the SVD filters were reduced after using the H-Scan technique compared to before using the H-Scan technique. This result further validates the sensitivity of the higher-order H-Scan technique to red blood cells and microbubbles. In particular, the introduction of the H-Scan technique allowed the filter to more accurately process and distinguish red blood cells and microbubbles from the target area, and thus effectively reduced the high-intensity tissue component. This not only demonstrates the ability of the H-Scan technology to improve the detection of subtle features in blood flow imaging, but also further emphasizes its advantages in enhancing imaging quality and accuracy.

In subsequent studies, the estimation of the threshold of the Casorati-SVD filter from the departure of the time singular values will be considered.

### Comparison with existing approaches

The main current approaches to blood flow ultrasound imaging using microbubbles are microbubble contrast-enhanced ultrasound (CEUS) imaging and ultrasound localization microscopy (ULM). The microbubble-based H-Scan method in this paper is compared to CEUS, ULM, and H-Scan-only techniques.

CEUS is a medical imaging technique that enhances the signal of ultrasound waves by injecting microbubbles into the flow of blood, which reflect off the ultrasound waves, resulting in improved contrast and detail in the image. The animal experimental part of the paper compares CEUS and microbubble-based higher-order H-Scan imaging using rat kidneys. The microbubble-based high-order H-Scan imaging not only retained the advantages of CEUS that could improve the contrast of blood flow, but also utilized the sensitivity of high-order H-Scan to red blood cells and microbubbles to further improve the quality of blood flow imaging. According to the paired *T*-test analysis results of animal experiments, SNR and CNR were significantly improved (*P* < 0.01).

ULM is a high-resolution ultrasound imaging technique. It combines ultrasound imaging and microbubble imaging to achieve sub-millimeter resolution in ultrasound imaging by precisely locating and imaging the echo signals of microbubbles. The algorithmic implementation of ULM depends on accurately detecting, localizing, and tracking the microbubbles in the blood flow to reconstruct the detailed structure of the blood vessels. Although ULM offers a significant advantage in imaging resolution, its implementation depends on sophisticated signal processing algorithms and high-performance ultrasound equipment. The calculation complexity of the algorithms for localization and tracking of microbubbles is high, and accurate reconstruction of microbubble position and motion is challenging. In contrast, the microbubble-based H-Scan technique has relatively low computational complexity, and it is easy to meet the requirements for an ultrasound probe with a moderate center frequency and a waveform that approximates the GH4 kernel.

The application of the H-Scan-only technique, which enhances Doppler imaging by utilizing Rayleigh scattering from red blood cells in the B channel, ignores the role and influence of microbubbles in H-Scan. In comparison, the work in this paper considers the specificity of the H-Scan for microbubbles and emphasizes the results of combining microbubbles with the H-Scan. Microbubbles are highly echogenic due to the large difference in acoustic impedance between the microbubbles and the surrounding tissue. Therefore, microbubbles used as contrast agents in ultrasound imaging can effectively improve the visualization of blood flow and tissue structure. High-order H-Scan technology can effectively detect not only red blood cells, but also a certain concentration of microbubbles. Therefore, microbubble-based H-Scan technology can further improve the quality of blood flow imaging over H-Scan-only technology.

### Limitations of microbubble-based H-Scan imaging technology

The microbubble-based H-Scan imaging technique also has some limitations in performing experiments. For example, the Casorati-SVD-based filter used for clutter filtering is extremely dependent on the selection of two eigenvalues (i.e., the thresholds for partitioning the tissue signal subspace from the blood flow signal subspace and the thresholds for partitioning the blood flow signal subspace from the noise signal subspace), and indeed, the prerequisites for the two thresholds are the assumptions that the tissue, the blood flow, and the background noise have completely different spatial and temporal characteristics, which is not the case for the practical application. Another limitation is that this paper ignores the problem of backscatter intensity attenuation that exists with increasing depth, which to some extent affects the imaging quality of deep tissues. In subsequent studies, the inclusion of an attenuation correction algorithm is considered to improve the imaging quality of micro-vessels. In addition, this paper lacks a comparison between the actual vascular distribution of the rat kidney and the ultrasound images, and future work considers anatomical analyses of the rat liver to visually verify the distribution of blood vessels.

## Conclusion

This paper proposes an ultrasound imaging method based on microbubble-enhanced H-Scan to further improve imaging of microvasculature. Using SNR and contrast-to-noise ratio (CNR), quantitative comparisons of power Doppler images before and after processing with H-Scan technology were conducted on rat liver data injected with microbubbles. The results demonstrate the effectiveness of the proposed method.
